# Preliminary Effectiveness of an In-Hospital Peer Support Program, Adolescent and Young Adult Hiroba, on Anxiety in Adolescent and Young Adult Patients with Cancer

**DOI:** 10.1089/jayao.2023.0065

**Published:** 2024-02-09

**Authors:** Takatoshi Hirayama, Rebekah Kojima, Ryoko Udagawa, Yuki Mashiko, Kazuko Matsumoto, Kyoka Ogata, Akie Shindo, Tomoko Mizuta, Yuko Ogawa, Ayako Kayano, Yuko Yanai, Hiroto Ishiki, Eriko Satomi

**Affiliations:** ^1^ Department of Psycho-Oncology, National Cancer Center Hospital, Tokyo, Japan.; ^2^ Department of Palliative Medicine, and National Cancer Center Hospital, Tokyo, Japan.; ^3^ Department of Pharmacy, National Cancer Center Hospital, Tokyo, Japan.; ^4^ Department of Palliative Care, National Center for Global Health and Medicine, Tokyo, Japan.

**Keywords:** adolescent and young adult, peer support, anxiety, distress, survivorship care, psychosocial support

## Abstract

**Purpose:**

Adolescent and young adult (AYA) patients with cancer have few opportunities to connect with patients of the same generation while hospitalized. Although anxiety is frequently reported by them, there are no reports on the psychological effectiveness of an in-hospital patient support program based on peer support. This study aimed to evaluate the effectiveness of a program, termed Adolescent and Young Adult Hiroba (AYA Hiroba), for anxiety in AYA patients with cancer.

**Methods:**

This single-center, prospective, observational study in 24 AYA patients with cancer was conducted at the National Cancer Center Hospital in Japan. The Hospital Anxiety and Depression Scale—Anxiety (HADS-A) was used to evaluate the primary outcome, anxiety. The Distress Thermometer (DT) was used to evaluate the secondary outcome, distress. The two outcomes were assessed before and after participation in AYA Hiroba. The Net Promoter Score (NPS) was used to evaluate satisfaction after participation in AYA Hiroba. Participants' free-text descriptions of the program were categorized according to similarities and differences.

**Results:**

The HADS-A and DT scores were significantly lower after the program than before (*p* < 0.001), as was the percentage of AYA patients with cancer with high distress (*p* = 0.04). The NPS was 27, which was lower than the value of 52 obtained in our previous study. Requests and suggestions to improve the program were grouped into three categories: content, facilitation, and online connection environment.

**Conclusion:**

This study suggests the preliminary effectiveness of the in-hospital peer support program for anxiety in AYA patients with cancer.

The Clinical Trial Registration number: UMIN000045779.

## Introduction

Approximately 87,000 and 20,000 adolescent and young adults (AYAs) in the United States and Japan, respectively, are new to cancer annually, representing to ∼4.5% and 2.3% of all people diagnosed with cancer.^1,2^

AYA patients with cancer reportedly find peer opportunities helpful.^3^ Support from other AYA patients with cancer is key to helping members of this age group manage their disease.^4^ AYA patients with cancer indicated that the opportunity to interact with other survivors of their own age was more valuable than the support they got from family and friends.^5^

To date, AYA support groups have incorporated a variety of peer-support formats such as weekly face-to-face meetings, online groups, weekend retreats, conferences, and therapeutic adventure trips. These activities help meet age-related developmental challenges and promote positive psychosocial growth.^6^ AYA patients with cancer should be given opportunities to attend these programs.

In Japan, different clinical departments care for these patients owing to the variety of primary cancer sites and the small number of AYA patients with cancer at each designated cancer center.^7^ AYA patients with cancer experience anxiety throughout their clinical course, including before diagnosis, when the diagnosis is shared, and during initial treatment.^8–10^ However, AYA patients with cancer currently have little opportunity to connect with their peers while hospitalized, so establishing an in-hospital peer support system was deemed necessary.

The National Cancer Center Hospital (NCCH) is a high-volume cancer center that treats >1000 new AYA patients with cancer annually. An in-hospital peer support program for AYA patients with cancer, named AYA Hiroba,^11^ was introduced in 2016. A multidisciplinary team supports the varied needs of this population. AYA Hiroba provides face-to-face get-together opportunities for AYA patients with cancer at NCCH. *Hiroba* means “a place or space for gathering” in Japanese.

However, AYA Hiroba was disrupted by the COVID-19 pandemic. Face-to-face meetings are considered a potential risk for SARS-CoV-2 transmission owing to patients gathering and talking in immediate proximity. To address safety concerns, AYA Hiroba has been conducted online since 2020. The positive effects of online peer support have been shown in patients with cancer, for example resulting in a lower prevalence of depression and reduced perceived stress.^12–15^ An online AYA community has been shown to be helpful for AYA patients with cancer in expressing feelings, sharing information, giving and receiving peer support, and coping with cancer.^16^ In Japan, the AYA generation uses the internet at a high rate, both via smartphones and personal computers.^17^ We named the online get-together opportunities for AYA patients with cancer, “Online AYA Hiroba.”^18^ Later, as the COVID-19–related restriction eased, peer support was provided in a hybrid manner, with both in-person and online events.

Anxiety is a commonly described problem among AYA patients with cancer.^8,19–22^ Although a systematic review and meta-analysis reported that peer support interventions significantly alleviated anxiety among patients with cancer,^23^ the psychological effectiveness of an in-hospital peer support program has not been described.

This prospective study aimed to evaluate the effectiveness of an in-hospital peer support program, AYA Hiroba, for anxiety in AYA patients with cancer at a designated cancer center in Japan.

## Methods

### Study design

This single-center, prospective, observational study was conducted to examine the preliminary effectiveness on anxiety of AYA Hiroba, an in-hospital peer support program held at the NCCH for AYA patients with cancer. In this study, the Hospital Anxiety and Depression Scale—Anxiety (HADS-A) was used to evaluate the primary outcome, anxiety. The Distress Thermometer (DT) was used to evaluate the secondary outcome, distress. The two outcomes were assessed before and after participation in AYA Hiroba. The Net Promoter Score (NPS) was used to evaluate patients' satisfaction after participation in AYA Hiroba. In addition, a free-text field for comments about the program was included in a questionnaire administered following participation.

This study was approved by our institutional review board (IRB No.: 2021-169).

### AYA Hiroba content

AYA Hiroba is held every month for 1 hour (from 3 p.m. to 4 p.m.). Participants are AYA patients with cancer aged 15–39 years who are inpatients or outpatients at NCCH. Patients attending the session are free to talk about any topic they choose. For instance, they might share movies they have recently seen, their preferred music, taking examinations, getting a job, human relationship, getting married, or managing the side effects of anticancer drugs, and the effects of treatment on reproductive function. Each session is attended by two facilitators from the AYA support team. Table 1 provides the facilitator's manual that was used. Each of the in-person participants was provided with a PC on site, and could use it to interact with the online participants.

**Table 1.** Manual for Facilitators

**(1) Program preparation**
(1) Leaflets with the program's URL and QR code were posted inside the hospital, including in the outpatient department.
Leaflets were also distributed to inpatients by AYA support team staff.
An application form was completed and submitted by patients wishing to participate in the program.
This form was distributed via the staff mailing list. The staff in charge of operations obtained each applicant's email address.^a^
The process of registering to participate was automated, but the appropriate staff could answer any questions.
To confirm whether each patient was eligible for participation in the AYA Hiroba program (i.e., whether or not they were an NCCH patient), the staff in charge of operations referred to the section entitled “With or without patient registration card for NCCH” on the application form.
Before the program began, the staff in charge of operations reviewed participant responses to an item on the application form that asked about topics that they would like to talk about.
^a^Patient email addresses were only used for contacting participants about the AYA Hiroba program and were strictly managed by the staff in charge of operations.
(2) Several days before the program, a program notice was sent by email to patients who had previously participated in the program and who opted to receive notifications from the program.
(3) Hospitalized applicants were informed that they could use Zoom to attend the program, which was held in person at the NCCH Patient Support Center^b^, for instance, if they had difficulty participating in person or needed private space because they were in a shared patient room.
^b^The Patient Support Center provides multifaceted support related to the cancer journey to patients with cancer and their families during treatment, discharge, and recurrence.
Either on the day of the session or the day before, suitable rooms were reserved for program operations at the Patient Support Center.
(4) The following were sent to participants by noon on the day of the session: (1) the URL for the session, (2) program rules, and (3) instructions on how to use Zoom.
A final meeting with facilitators was held 20 minutes before the session, to make sure the session started on time.
**(2) How to conduct the meeting**
(1) Opening remarks:
“Hello everyone. Can you activate your camera?”
“Several more people are going to participate. Please wait a few minutes.”
Or “There may be some participants joining later. It is now time to start the meeting.”
Confirm that the usernames of the participants on Zoom are the names that they want to use during the sessions.
Some cameras cannot be activated in certain Wi-Fi network settings—this information should be shared with the participants.
If there is anything unusual, such as an observer, let them know.
(2) Describe the rules of the meeting (using slides through a shared screen):
“There are some rules for the AYA Hiroba program. I will present these rules and the agenda for this session.”
(3) Provide an introduction and other information about each participant, including the participants' usual activities, favorite things, concerns, or interests.
“Let's begin by introducing yourselves. Tell us three things about yourself: your name, type of cancer and ongoing treatment, and your favorite things.”
(4) Have an open conversation or give the participants some choices for discussion topics.
Engage in an open discussion or give the participants some choices for discussion topics (for instance, using a flip chart containing 10 conversation topics that was prepared in advance by the AYA support team).
If there is little open discussion, mention the topics listed by participants on the application form as those that they would like to discuss.
Flip chart contents (some related to illness or treatment, others to daily life):
(1) What do you do when you have free time?
(2) What do you want to recommend to everyone?
(3) How do you cheer yourself up when you feel depressed?
(4) If you win the lottery, what will you buy?
(5) What is one thing you couldn't stop doing within the last year?
(6) Who did you first tell about your illness?
(7) Who can you tell about your illness? Who can't you tell?
(8) What has changed since you got sick? What hasn't changed?
(9) How do you change your mood in the hospital?
(10) Do you have any question that you would like to ask others?
Provide a 5-minute warning before the end of the session (this can be done via chat, if needed).
(5) Ask the participants for brief feedback before ending the session.
“It is almost time to end the session. Can you give us a short comment about this session?”
(It will run more smoothly if the facilitator calls on the participants in order.)
(6) Ask the participants to complete the post-meeting questionnaire (tell the participants that a survey link will be sent after the session).
“To use your feedback to improve meeting facilitation in the future, we will send you a questionnaire via e-mail after this session. We would grateful if you answered it. Thank you in advance for your cooperation.”
(7) Inform the participants of the scheduled date of the next session.
“The next meeting will be held from 3 p.m. to 4 p.m. on MMDDYYYY.”
For participants who want to receive notice of the next meeting (by choosing “Yes” on the questionnaire), inform them that the notice will be delivered several days before the next session.
“If you choose to receive notifications about subsequent sessions (by marking ‘Yes' on the questionnaire), we will inform you with a notice delivered several days before. Thank you in advance for your kind understanding.”
(8) Close the meeting.
“That is all for today. Thank you for your attendance. Please click on the Leave button to disconnect.”
Facilitators will leave the session after seeing the participants sign off.
**(3) Facilitators' roles (session chair and co-chair)**
Preferably, the two facilitators authorized as hosts should be in the same room.
Include additional information, such as name, chair/co-chair, and title, next to these individuals' Zoom usernames.
Make prominent gestures and reactions (e.g., nodding, empathizing, laughing, being surprised) and actively use the reaction emojis on Zoom. Encourage participants to do so as well.
Chair:
Participate using earphones with a microphone, and mute the microphone when the co-chair is speaking to avoid microphone feedback.
Presents topics to discuss, announces how much time is left, and decides who should speak next, considering the overall course of the meeting.
Be sure to give some brief feedback after each participant's comments before moving on to the next participant. (It's not a critique, so be careful not to make it too long.)
When it is appropriate, ask for introductions from those who joined in the middle of the session.
Briefly introduce current participants to those who have just joined.
In an online meeting, participants tend to become silent because they are hesitant to talk. To avoid this silence, the chair may pose questions to specific participants, such as “Do you feel similarly about …?” to ensure that the meeting flows smoothly.
Co-Chair:
Participate using earphones with a microphone, and mute the microphone when the chair is speaking to avoid microphone feedback.
Respond to any e-mail messages or chat inquiries from the participants.
Update late-joining participants via chat about conversations that had already taken place.
Pay careful attention to participants in the Patient Support Center.
Provide necessary information to the participants via chat or through on-screen messages, including the amount of time remaining.
**(4) Actions that should be taken by the chair or co-chair during the meeting:**
If a participant recommends any folk remedies,
→ (Sample reply) the facilitators respond: “You should consult with your primary doctor prior to using this because it could have a negative physical impact on some patients.”
If a participant is speaking for too long,
→ (Sample reply) The facilitators state: “Mr./Ms. A has provided his/her thoughts on B. Is there anyone else who feels the same way or would like to share their thoughts?”
If a participant seems to be acting strangely, for example, if the facilitators are worried about the physical condition of the participant,
→ (Sample reply or chat) The facilitators ask: “Are you all right, Mr./Ms. C?”

AYA, adolescent and young adult; NCCH, National Cancer Center Hospital.

There were three participation rules: (1) “You will never have to tell other participants about anything you feel uncomfortable sharing” (2) “Do not disclose anything you heard or known from this session,” and (3) “Do not criticize other participants. Respect others.” The facilitators adhered to the following policies listed in the facilitator's manual: (1) be courteous and follow the rules listed in Table 1; (2) try to ensure that everyone feels comfortable and speaks to the group as a whole; (3) take off hospital coats; (4) minimize feedback and respond with constructive “I” messages (e.g., say “I feel” to gently point out something); and (5) elicit questions and opinions from participants.

### Study participants

The study participants were AYA patients with cancer (aged 15–39) who participated for the first time in the AYA Hiroba peer support program held at the Patient Support Center of the NCCH from November 2021 to April 2023.

### Sample size

Base on a previous study,^24^ the sample size was calculated with the one-tailed test using the following: the mean value of two groups with 1.41 points as the Minimal Clinically Important Difference (MCID) of the HADS-A; standard deviation (SD) 2.65; *α* = 0.05; and power (1−*β*) = 0.80. The calculation resulted in the recruitment of 24 participants.

### Procedures

In routine clinical practice, AYA patients with cancer who wish to participate in AYA Hiroba apply using an application form on the NCCH website. On the form, they specify whether they want to participate in the program in person or online. In this study, therefore, the names and email addresses obtained from this application form were used to recruit study participants. Those enrolled in the study were sent an email announcing confirmation of participation in AYA Hiroba, along with an explanatory document about the study and a web address that provided access to the preprogram questionnaire. After the AYA Hiroba program concluded, a web address with a postprogram questionnaire was sent to participants, and the study was considered complete when they responded. The HADS-A and DT were used to evaluate AYA patients with cancer before and after participation in AYA Hiroba.

### Analysis

Background information on participants was extracted from medical records (medical record number, date of birth, gender, study registration number, disease name, stage of illness, treatment history, treatment details, marital status, parental status, residential status, employment/school status, etc.). Data were analyzed using descriptive statistics.

The primary outcome was anxiety, determined by the HADS-A before and after participation. The HADS-A^25,26^ is a self-administered scale that measures mental status related to anxiety. It consists of seven questions, each of which is answered using a 4-point scale, resulting in a score of 0–3. Higher scores indicate worse mental status.

The secondary outcomes were distress, measured by the DT before and after participation, and the NPS. The DT^27,28^ is a self-administered questionnaire used to measure the emotional distress experienced by patients with cancer during the week before questionnaire completion. It is rated on an 11-point scale, resulting in a score of 0–10. Higher scores indicate more painful feelings. In this study, DT scores were analyzed with the paired *t*-test. A DT score ≥5 was defined as high distress, because a score of 5 was previously shown to result in the best clinical screening cutoff in AYA patients with cancer.^20^ The percentages of AYA patients with cancer and high distress (number of patients with a DT score ≥5 points)/(number of patients who reported any DT score) before and after receiving the support program were compared using the chi-square test.

The NPS,^29^ a single-item measure of consumer satisfaction, was used to assess trial performance among conditions. This tool has been implemented as an overall measure of patient experience with health care delivery. Patients were asked, “How likely would you be to encourage another patient like you to participate in this program?” Participants with scores of 0–6 were considered detractors; 7–8, passive; and 9–10, promoters. The percentage of promoters minus the percentage of detractors was calculated. NPS could range between −100 and 100.

Free-text descriptions of the program were classified based on similarities and differences using the KJ method^30^ by a psychotherapist and a psychiatrist.

## Results

### Participants' demographic characteristics

**FIG. 1.**
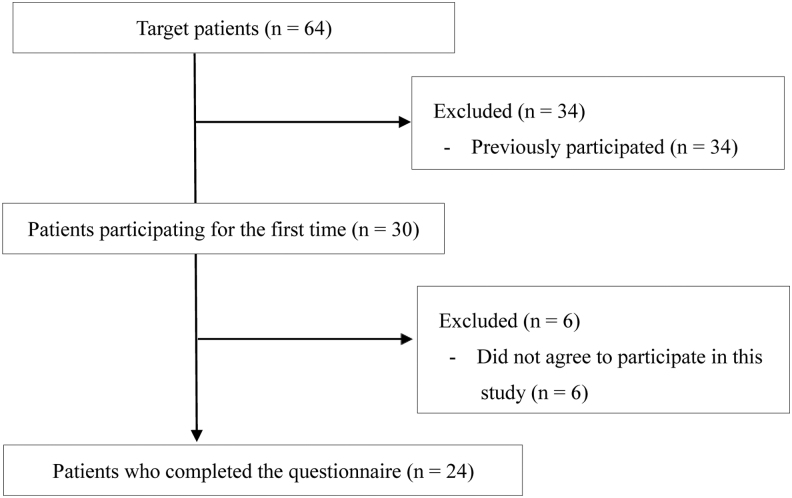
Patient flow.

Patient flow is given in Figure 1. Of 64 participants, 30 were first-time participants. Six patients did not agree to participate in this study. On average, there were 3.6 participants at each session.

The demographic characteristics of participants who completed this study are given in Table 2. There were 13 females and 11 males, with an average age of 30.0 years (SD ±5.3 years). Eighteen participants (75.0%) were outpatients and 12 (50.0%) took part in the program online. Six participants (25.0%) had graduated from college, and 14 (58.3%) were employed. Primary cancer sites included bone and soft tissue (*n* = 6, 25.0%), hematologic (*n* = 5, 20.8%), urogenital system (*n* = 3, 12.5%), digestive system (*n* = 3, 12.5%), lung (*n* = 2, 8.3%), and others (*n* = 5, 20.8%). The most common stage at diagnosis was stage IV (*n* = 4, 16.7%), followed by stage I (*n* = 3, 12.5%). The most common treatment setting was curative (*n* = 18, 75.0%). The most common ongoing anticancer treatment type was chemotherapy (*n* = 10, 41.7%), followed by surgery (within 1 month before program participation) (*n* = 4, 16.7%). Thirteen participants (54.2%) had spouses, and 7 (29.2%) had children. Nineteen (79.2%) were living with at least one other person.

**Table 2.** Respondents' Demographic Characteristics (*n* = 24)

	No. of patients	Percentage of patients (%)
Age, years (mean 30.0 ± 5.3)		
15–19	1	4.2
20–24	2	8.3
25–29	8	33.3
30–34	9	37.5
35–39	4	16.7
Gender		
Female	13	54.2
Male	11	45.8
Inpatient or outpatient		
Outpatient	18	75.0
Inpatient	6	25.0
Online or on-site participation		
Online	12	50.0
On-site	12	50.0
Education status		
College graduate	6	25.0
High school graduate	3	12.5
Graduate school graduate	1	4.2
Junior high school graduate	1	4.2
Unknown	13	54.2
Social status		
Employed	14	58.3
Student	5	20.8
Unemployed	5	20.8
Cancer type		
Bone/soft tissue	6	25.0
Hematological	5	20.8
Urogenital system	3	12.5
Digestive system	3	12.5
Lung	2	8.3
Brain	1	4.2
Breast	1	4.2
Gynecological system	1	4.2
Head and neck	1	4.2
Others	1	4.2
Disease stage		
IV	4	16.7
I	3	12.5
II	1	4.2
III	1	4.2
Other	5	20.8
Hematological cancer		
Unknown	10	41.7
Therapeutic purpose		
Curative	20	83.3
Palliative	4	16.7
Cancer treatment history^a^		
Chemotherapy	16	66.7
Surgery	13	54.2
Radiation	7	29.2
Transplantation	3	12.5
None	2	8.3
Cancer treatment in progress^a^		
Chemotherapy	10	41.7
Surgery (within 1 month before program participation)	4	16.7
Radiation	1	4.2
None	10	41.7
Has spouse or partner		
Yes	13	54.2
No	11	45.8
Parent		
No	17	70.8
Yes	7	29.2
Living situation		
Living with someone else	19	79.2
Living alone	5	20.8
Religion		
No	24	100
Yes	0	0

^a^ Multiple responses allowed for each patient.

The frequently discussed topics in AYA Hiroba were getting a job, human relationship, and how to manage the side effects of anticancer drugs.

### The HADS-A score

The HADS-A score was significantly lower after the program than before (*p* < 0.001) (Table 3). The mean difference in the HADS-A score before and after the program was >1.41 points, which we had predefined as the MCID.

**Table 3.** Score Differences Before and After the Program

	Before	After	*t*	Cohen's* d*	*p*
	*M *(SD)	*M *(SD)			
Hospital Anxiety and Depression Scale—Anxiety	9.4 (5.6)	7.0 (5.5)	3.9	0.79	<0.001
Distress Thermometer	5.3 (2.5)	3.5 (2.6)	4.3	0.87	<0.001

*M*, mean; SD, standard deviation.

### The DT score

The DT score was significantly lower after the program than before (*p* < 0.001) (Table 3), as was the percentage of AYA patients with cancer and high distress (8 of 24 [33.3%] vs. 15 of 24 [62.5%], respectively; *p* = 0.04).

### The NPS and free-text descriptions of the program

Eight, 12, and 2 participants reported an NPS of 9–10, 7–8, and 0–6, respectively.

Two participants did not report NPS. Thus, the overall NPS was 27, which was lower than the value of 52 obtained in our previous study.^18^ The common demographic characteristics of the two participants who reported an NPS of 0–6 were male outpatients whose therapeutic purpose was curative.

Requests and suggestions to improve the program were grouped into three categories: content, facilitation, and online connection environment (Table 4).

**Table 4.** Questionnaire Answers

Please write down your requests or suggestions for improvement with respect to AYA Hiroba, if applicable.
(Content) - When I imagined participating during my own battle with the disease, sometimes I didn't have the energy to get out of bed, so I thought it would be a good idea to have more participants who just listen. I thought that even if I didn't have the energy to speak, just listening to the cheerful atmosphere would cheer me up. - I was able to talk to people of my generation who have been patients longer than I have, and it was comforting to know that I am not alone and that I can continue to enjoy my life. I am not alone and I will be able to enjoy my life in the future. Thank you very much. - If the AYA Hiroba party is held after COVID-19 ends, I would like to attend. - It was my first time attending this meeting and it was really energizing and encouraging to hear from actual survivors of the AYA generation. I would definitely like to attend the next one. Thank you so much. - I wanted to hear from doctors and psychologists as well as patients. - I thought I could have answered the questions more smoothly if I had known the topic beforehand. - I had a good time. - It was easy to talk with only women. I thought it would be good to have separate days for different genders. (Facilitation) - This was my first time attending, but the facilitator, a pharmacist, was upbeat and spoke to everyone equally, so I was able to hear from many people. It was very informative and enjoyable. Thank you very much. - Since it was my first time attending, I did not usually know what was going on, but the conversation was centered on the participants, and I was concerned that those who were struggling or in need might be happy to have attended. I know it's very difficult, but I felt the facilitator should pay a little more attention to each individual. (Online connection environment) - Consider installing a microphone or other equipment because it is sometimes difficult to hear other people when they are wearing a mask. - The microphone in the hospital was a little far away. - It would be easier for participants on site to talk without worrying about noise, etc., if they participated at a single terminal device.

## Discussion

This study is the first to verify the preliminary effectiveness of an in-hospital peer support program on anxiety in AYA patients with cancer.

Regarding the background of the participants, the recruitment of adolescent patients aged 15–24 years was a challenge because they comprise <10% of the patient population with cancer at the NCCH. However, age 15–19 years was reported to be associated with mental health care use and adolescent patients have psychological needs.^31^ There is a need to identify factors that are barriers to adolescent participation and to find effective recruitment methods. In this study, approximately half of the participants were male. In previous online studies^16,32,33^ and in a study on a face-to-face AYA Hiroba program,^11^ the proportion of male participants was low.

Thus, online^18^ or hybrid sessions may be more accessible to male patients in Japan for peer support participation. Outpatients and those participating online each accounted for more than half of the total (with overlap). This suggests that program participation is facilitated by being able to engage with the program via the internet from outside the hospital, such as from home. This result is similar to a previous cross-sectional study in which AYA patients with cancer indicated that the main barrier to accessing peer support was the lack of convenience of in-person support groups.^34^ The common demographic characteristics of the two participants who reported a low NPS were male outpatients whose therapeutic purpose was curative. Although the sample size is small, it should serve as a reference for future improvements of AYA Hiroba program.

The three most common cancer types in AYA patients with cancer in this study are known to have a high rate of anxiety during treatment, for a number of reasons. In bone or soft tissue cancer, the risk of chemotherapy affecting fertility has been shown.^35,36^ Patients with hematological cancer, particularly those undergoing hematopoietic stem cell transplantation, often endure longer-than-average and therefore more intrusive treatment interventions, which can lead to a greater likelihood of side effects and late effects. Unmet psychological needs have been reported in many AYA patients with cancer who receive hematopoietic stem cell transplantation.^37^ In urologic cancers, the cancer directly affects fertility.

Stage IV was the most common disease stage in this study, suggesting that the possibility of online participation made the program more accessible to patients with advanced cancer, many of whom have difficulties with activities of daily living.^38^ The most frequent treatment type was chemotherapy, indicating that patients receiving chemotherapeutic regimens can participate online from their hospital rooms or homes, even if they are experiencing side effects.

The primary outcome, anxiety, was significantly reduced after the program, as measured by the HADS-A score. The mean difference in the HADS-A between before and after the program exceeded the predefined MCID, suggesting a preliminary effect of the program on anxiety. Our findings are consistent with that of a systematic review and meta-analysis.^23^

The secondary outcome, distress, was also significantly lower after the program, as determined by the DT. The effect size of this study (Cohen's *d* = 0.87) was larger than that of an observational study in Japan of a psychosocial support program for AYA patients with cancer evaluated using the DT and Problem List—Japanese version (Cohen's *d* = 0.21).^22^ This suggests that peer support reduces distress in AYA patients with cancer to a larger extent than psychosocial support. The percentage of AYA patients with cancer who reported high distress before program participation in this study (62.5%) was higher than that of AYA patients with cancer before the psychosocial support program mentioned previously (33.5%),^22^ suggesting that AYA patients with cancer seeking peer support experience pronounced distress.

On the contrary, the NPS was lower than that measured after the Online AYA Hiroba program mentioned previously.^18^ The integration of in-person and online patient participation in the hybrid program in this study was operationalized using a trial-and-error process. As noted by responses in the free-text portion of the questionnaire, facilitation and the online environment need to be strengthened. In the end, the facilitator manuals were created as given in Table 1, but the low NPS may have arisen because the hybrid nature of the program (i.e., both online and in-person) made it more complicated to manage than the online-only program in a previous study.^18^ This may have had an especially negative effect on the experiences of individuals who took part in-person.

There are some limitations to this study. First, it was a single-center study with few participants. Generalizing these results to other institutions might be difficult. Second, this program was only for NCCH patients. Although a wide range of outpatients can participate online, including those at other hospitals, for the purposes of this study it was important to be able to verify whether an online outpatient was actually a patient with cancer and a member of the AYA generation, and this was possible only for NCCH patients. Third, the MCID of the HADS-A used for the sample size calculation was defined in chronically ill patients,^24^ because that in patients with cancer has not been reported yet. Finally, all participants were treated or pre-treatment. The level of interaction between patients that is required within medical institutions is an area for further study, as it is expected that interactions between long-term survivors and post-treatment patients take place inside and outside of the hospital, for instance, in patient groups.

Despite these limitations, the results of this study and the development of facilitator handbooks can help develop an in-hospital peer support program to reduce anxiety in AYA patients with cancer at other hospitals. We intend to further develop this program to increase the opportunities of AYA patients with cancer to interact with peers and to improve their quality of life.

## Conclusion

This study suggests the preliminary effectiveness of an in-hospital peer support program, AYA Hiroba, for anxiety in AYA patients with cancer.
